# Single-Cell Analysis: A Method for In-Depth Phenotyping of Cells Involved in Asthma

**DOI:** 10.3390/ijms252312633

**Published:** 2024-11-25

**Authors:** Daniel Rodríguez-González, Gema Guillén-Sánchez, Victoria del Pozo, José Antonio Cañas

**Affiliations:** 1 Instituto de Investigación Sanitaria Fundación Jiménez Díaz (IIS-FJD), Universidad Autónoma de Madrid (UAM), 28040 Madrid, Spain; daniel.rodriguezg02@estudiante.uam.es (D.R.-G.); gema.guillensanchez@usp.ceu.es (G.G.-S.); jose.canas@fjd.es (J.A.C.); 2 Centro de Investigación Biomédica en Red (CIBER) de Enfermedades Respiratorias (CIBERES), 28029 Madrid, Spain; 3 Medicine Department, School of Medicine, Faculty of Medicine, Campus of Medicine, Universidad Autónoma de Madrid (UAM), 28029 Madrid, Spain

**Keywords:** single-cell RNA-seq, asthma, immune cells, allergic diseases

## Abstract

Asthma is a chronic inflammatory lung disease with high prevalence, making it one of the most common chronic conditions worldwide. Its pathophysiology is influenced by a range of genetic and environmental factors, resulting in a complex and heterogeneous disease profile. Asthma is primarily associated with a type 2 (T2) immune response, though non-T2 endotypes also contribute to disease pathology. Generally, asthma is characterized by the infiltration and activation of various cell types, including dendritic cells, eosinophils, innate lymphoid cells, lymphocytes, mast cells, and neutrophils, which participate in T1, T2, and T17 immune responses. Despite advances in understanding, many questions remain unresolved. Therefore, emerging omic techniques, such as single-cell RNA sequencing (scRNA-seq), offer novel insights into the underlying mechanisms of asthma and the roles of these immune cells. Recent scRNA-seq studies in asthma have identified multiple novel immune cell subtypes and clusters, suggesting their potential functions in disease pathology. The rapid advancement of scRNA-seq technology now enables in-depth investigation of individual cells within tissues, allowing for precise cell-type classification and detailed molecular profiling. Nonetheless, certain limitations persist, which require further refinement in future studies.

## 1. Introduction

### 1.1. Pathophysiology and Treatment of Asthma

Asthma is a chronic inflammatory disease that affects the airways [1], and its prevalence is increasing, making it one of the most common chronic diseases [2]. The main symptoms are cough, shortness of breath, and wheezing [1]. Multiple factors, both genetic and environmental (such as cold air, exercise, infections, mutations, and tobacco smoke), contribute to the development of this condition. These factors lead to airway obstruction and trigger hyperresponsiveness of the immune system (both innate and adaptive). This results in edema, mucus secretion, and even permanent structural remodeling of the airways. The latter is caused by the infiltration and activation of various cell types, including dendritic cells (DCs), eosinophils, innate lymphoid cells (ILCs), lymphocytes, mast cells, and neutrophils. These cells are involved in T1, T2, and T17 immune responses, along with the genetic predispositions mentioned earlier [2].

For many years, experts have classified asthma into two main types of phenotypes (observable characteristics that result from a combination of hereditary and environmental influences): allergic and non-allergic asthma. Allergic asthma typically begins in childhood and is associated with a T2 immune response, such as in cases of allergic dermatitis or rhinitis. T helper type 2 (Th2) cells release cytokines, including IL-4, IL-5, IL-9, and IL-13. These cytokines contribute to the accumulation of eosinophils, excessive mucus production, and the synthesis of immunoglobulin E (IgE) by B lymphocytes. Allergic asthma is often linked to eosinophilia, rhinosinusitis, and the presence of nasal polyps [1]. On the other hand, non-allergic asthma tends to appear in later stages of life and is more prevalent in women [3], smokers, and individuals who are overweight or obese [4].

Experts now agree that asthma should be classified based on its endotypes rather than its phenotypes [5]. The biological mechanisms underlying the development of the disease provide a more accurate framework for classification than clinical, demographic, or pathophysiological characteristics [6]. Understanding these mechanisms allows physicians to implement personalized therapies and optimize disease management. Nevertheless, it is important to note that some patients may not fit neatly into any one endotype, and there is often overlap between endotypes [7].

Currently, asthma is classified into two primary endotypes, as it takes into account pathophysiological mechanisms at the cellular and molecular levels: type 2 (T2) and no type 2 (non-T2) [8]. The T2 endotype is characterized by eosinophilia and is associated with cytokines such as IL-4, IL-5, and IL-13 and can be allergic or non-allergic. The non-T2 endotype, on the other hand, is for non-eosinophilic asthma patients and encompasses a more complex group of patients who do not exhibit T2 characteristics. This classification, which is based on pathophysiological mechanisms, helps in assessing responses of patients to treatments such as biologics or corticosteroids [1]. Therefore, accurate diagnosis is crucial for establishing patient-specific therapies to control symptoms. It is also important to consider that some patients may have overlapping diseases, such as chronic obstructive pulmonary disease (COPD). These patients experience more frequent exacerbations, lower quality of life, and higher morbidity compared to those with only one of these conditions [9]. Additionally, comorbidities like rhinosinusitis or gastroesophageal reflux disease (GERD) can coexist with asthma and exacerbate its symptoms. Therefore, proper identification and treatment of these conditions are essential [10].

Biomarkers are limited in their usefulness and apply only to certain asthma endotypes. Some of the key biomarkers include eosinophils, IgE, leukotrienes, neutrophils, periostin levels, and fractional exhaled nitric oxide (FeNO) [2]. The T2 endotype is marked by elevated eosinophil counts in both the airways and blood, as well as high IgE levels. In contrast, patients with the non-T2 endotype typically have normal eosinophil counts but elevated neutrophil levels [11].

The primary goal of asthma treatment is to control symptoms and improve quality of life. Non-pharmacological therapies for allergic asthma include avoiding allergens, warming up before and after exercise, improving physical fitness, and training in warm, humid environments [12]. Otherwise, pharmacological treatments include short-acting beta2 agonists (SABAs), inhaled corticosteroids (ICS), long-acting beta2 agonists (LABAs), and biological drugs such as benralizumab or mepolizumab for severe cases [10].

### 1.2. Single-Cell Analysis

The single-cell technique sequences the genome or transcriptome of individual cells. It provides genomic, transcriptomic, and multi-omic data to compare the various cell types within a sample. In other words, this method focuses on studying individual cells and their behavior rather than analyzing the sample as a whole. This is made possible by the phenotyping capability of single-cell analysis. The technique helps us understand the complexity and heterogeneity within cell populations. It generates transcriptional profiles that offer highly valuable genetic-level information, identifying which genes are being expressed, in what quantities, and how expression varies between cells. By gaining detailed insights into cellular behavior, single-cell analysis significantly enhances the precision of diagnosis and personalized treatments, which is the direction modern medicine is heading. Furthermore, it allows for the study of rare cells, which are often difficult to differentiate, thereby improving prognosis through earlier diagnosis.

**Figure 1:**
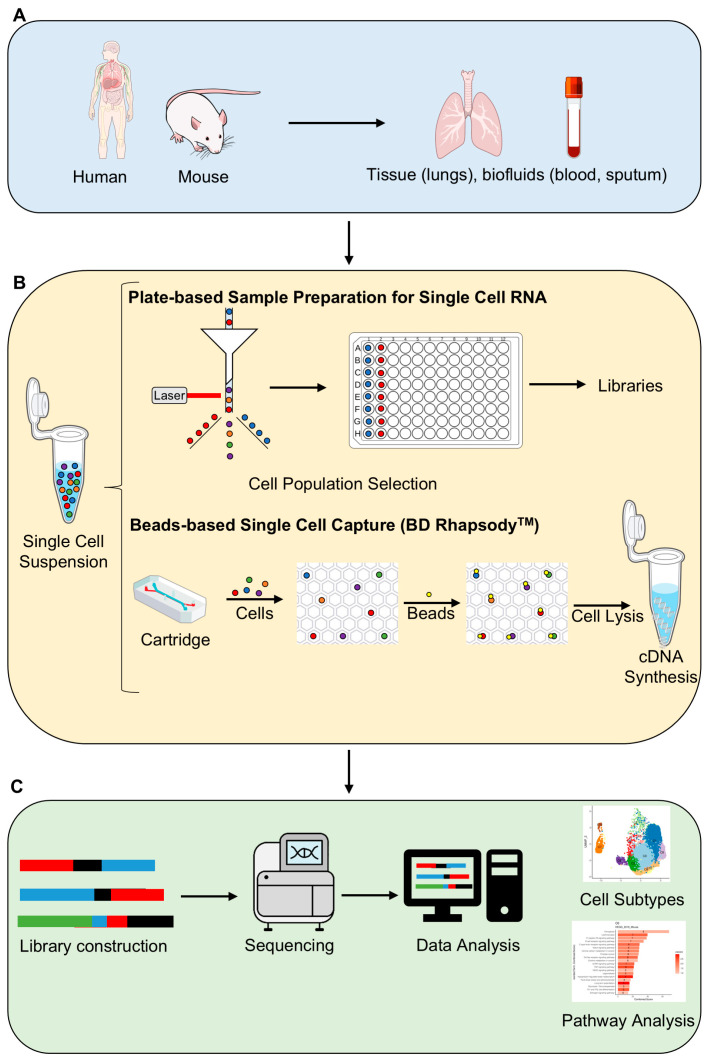
Steps of single-cell RNA-seq technique and analysis. In general, the workflow of single-cell sequencing includes the following steps: (**A**) Sample collection and preparation: initial collection and processing of the sample. (**B**) Isolation of single cells and cDNA synthesis: single cells can be isolated using various methods, such as plate-based sample preparation or bead-based single-cell capture. (**C**) Library preparation, sequencing, and data Analysis: construction of libraries, followed by sequencing and subsequent data analysis using bioinformatics tools.

The general workflow of scRNA-seq analysis consists of the following key steps (Figure 1): (i) single-cell isolation: cells are disaggregated, and a lysis solution is added to break the cell. A ribonuclease inhibitor is included to prevent RNA degradation and ensure its stabilization. Different methods for single-cell isolation are available: fluorescence-activated cell sorting (FACS), magnetic-activated cell sorting, microfluidic systems, and laser microdissection; (ii) nucleic acid isolation: the lysis solution contains oligo (dT) primers, which specifically bind to mRNA. This allows mRNA to be converted into complementary DNA (cDNA) through a reverse transcription reaction; (iii) amplification: the resulting cDNA is amplified using PCR to generate sufficient material for downstream analyses; (iv) sequencing: libraries are prepared similarly to classical RNA-seq protocols, enabling the sequencing of amplified cDNA; (v) data analysis: this complex and evolving step involves multiple specialized tools and is divided into four main phases: data acquisition (includes alignment or mapping, quantification, and de-duplication of sequencing reads), data cleansing (entails quality control, normalization, and imputation to ensure the reliability of the data), cell assignment (involves clustering, classification, and sorting of single cells into distinct groups based on their profiles), and gene identification (focuses on gene tagging, differential expression analysis, and identifying patterns of gene expression).

In recent years, costs have decreased, and there have been numerous advancements in amplification, data analysis, and sample collection. However, further improvements are still needed, particularly in amplification efficiency, cost reduction (as it remains expensive), RNA loss prevention, and information accuracy (since the data can still be incomplete or limited) [13]. This technique offers several advantages over traditional RNA sequencing, providing a deeper and more detailed understanding of cellular heterogeneity: (i) resolution of cellular heterogeneity: single-cell RNA sequencing (scRNA-seq) can reveal rare or previously uncharacterized subpopulations of cells that may be overlooked in bulk sequencing; (ii) identification of new cell types and states: scRNA-seq enables the discovery of novel cell types, states, or transitional stages that may not be evident in bulk RNA sequencing; (iii) deeper insight into cellular function: by profiling gene expression at the single-cell level, scRNA-seq provides a more nuanced understanding of cell-specific functions, regulatory pathways, and cellular interactions; and (iv) tracking cellular responses over time: scRNA-seq is particularly powerful in tracking cellular responses during developmental processes, differentiation, or reactions to treatment over time. Moreover, although this technique traditionally requires fresh samples, some studies have demonstrated that it can also be applied to cryopreserved samples [14]. As a result, single-cell analysis is being applied in numerous fields, including cancer research, biomarker discovery, therapeutic target identification, the identification of new cell types or regulatory processes, immunology, neurology, microbiology, organ development, and reproductive medicine.

Therefore, scRNA-seq is a powerful tool for identifying cellular clusters and deciphering the mechanisms underlying asthma, such as the role of *Creb5*, which has been shown to promote exacerbations induced by allergic reactions and viral infections [15]. A recent study using scRNA-seq to analyze endobronchial biopsy specimens from asthma patients and healthy controls similarly identified transcriptionally distinct basal epithelial subsets [16]. These subsets expressed IL-33, TSLP, and an IL-4/IL-13 gene signature, underscoring the significance of these airway progenitor cells and suggesting that pathobiological mechanisms detected in the upper airway may provide insights into lung disease.

**Figure 2:**
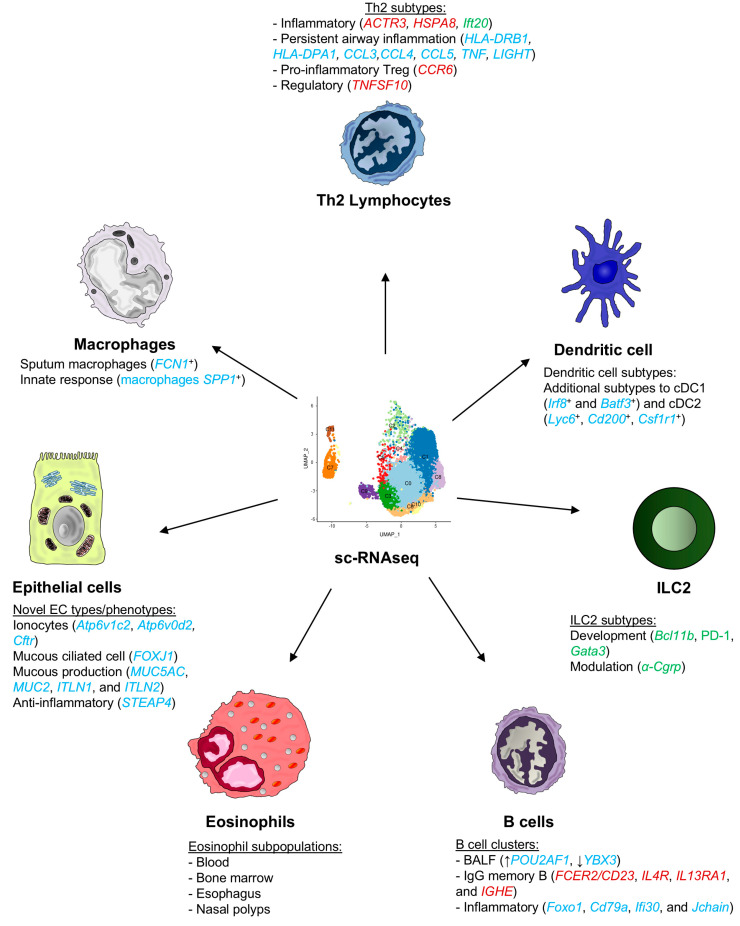
Single-cell analysis of key cell types involved in asthma pathogenesis. Single-cell RNA sequencing (scRNA-seq) studies have been performed on various cell types implicated in asthma pathogenesis, including T and B lymphocytes, dendritic cells, eosinophils, epithelial cells, type 2 innate lymphoid cells (ILC2), and macrophages. These studies have identified previously unknown genes that may play an important role in asthma development, and their analysis has led to the establishment of several new subtypes or clusters of these cells, contributing to the understanding of the underlying mechanisms of asthma. Human genes are written in italics and uppercase, whereas mouse genes are written in italics and lowercase, in accordance with international nomenclature standards. Additionally, each gene is color-coded based on the cell and origin source: red for blood, blue for airway and lung tissue, and green for other sources (e.g., intestine, spleen, bone marrow, etc.).

In this review, we will compile all the information related to scRNA-seq studies on the main cells involved in the pathogenesis of asthma (Figure 2).

## 2. ScRNA-Seq Analysis of Th2 Lymphocytes

In the context of a T2 immune response, particularly in asthma, one of the key cell types involved is T lymphocytes, specifically Th2 lymphocytes. In allergic asthma, Th2 cells play a crucial role in recruiting and activating other cells involved in the T2 inflammatory response, including eosinophils, mast cells, and IgE-producing B cells [17]. Given their critical role in allergy and asthma, an in-depth study of these cell types provides valuable insights into the mechanisms underlying asthmatic pathology, aiding in a deeper understanding of the disease.

In recent years, several studies using scRNA-seq have revealed new T cell subtypes and their roles in asthma pathogenesis [18]. Seumois and colleagues demonstrated that human Th lymphocytes and regulatory T cells (Treg) responding to house dust mite (HDM) allergens show significant heterogeneity, with different subsets varying both quantitatively and qualitatively in individuals with HDM-allergic asthma [19]. Interestingly, a greater number of HDM-reactive Th and Treg cells expressing the interferon response signature were present in asthmatic individuals without HDM allergy, compared to those with HDM allergy. These cells exhibited enriched expression of *TNFSF10*, suggesting that these subsets may suppress allergic responses, potentially explaining why only a small percentage of individuals develop T2 responses to nearly ubiquitous allergens. Similarly, scRNA-seq analysis identified a subset of immune-activating effector T cells that distinguish patients with asthma following allergic rhinitis, which may play a key role in post-allergic rhinitis asthma [20]. This cluster showed upregulation of *ACTR3* and *HSPA8* genes in the blood of asthmatic patients compared to healthy individuals. In an asthmatic mouse model, Jeong et al. identified novel proteins involved in CD4^+^ T cell regulation, showing that *Ift20* was upregulated in stimulated CD4^+^ T cells compared to unstimulated cells [21]. CD4^+^ T cells were stimulated with anti-CD3 and anti-CD28 antibodies, showing that Ift20 (a protein involved in transporting to nonmembrane-bound particles [22]) was upregulated in stimulated cells compared to unstimulated. Moreover, they found a direct protein–protein interaction between Ift20 and Tsg101 (endocytosis-related protein), and this interaction increase the phosphorylation of the Akt-mTOR mTor pathway (a key signaling pathway involved in asthma inflammation [23]).

Recently, a study performed in severe, uncontrolled asthmatic patients has revealed a notable heterogeneity in the CD4^+^ T cell populations present in the airways of these patients, with the tissue-resident memory T cells being the most abundant in the airways of severe asthmatics [24]. Moreover, this cell subset showing transcripts associated with T cell receptor activation (*HLA-DRB1*, *HLA-DPA1*) and cytotoxicity (*GZMB*, *GZMA*) and, after stimulation, elevated levels of transcripts encoding pro-inflammatory non-Th2 cytokines (*CCL3*, *CCL4*, *CCL5*, *TNF*, *LIGHT*) were detected, which may contribute to persistent airway inflammation and remodeling. Moreover, Liu et al. discovered reduced cuproptosis (a newly identified form of cell death) in CD4^+^ T cells from children with allergic asthma, suggesting that these immune cells may be closely associated with cuproptosis in allergic asthma development [25]. Other studies using scRNA-seq on murine models of allergic airway inflammation revealed that T cells from the lungs of mice sensitized with *Alternaria alternata* showed genetic signatures of elevated oxidative and glucose metabolism, indicating that T cells upregulate metabolic markers during airway inflammation [26].

In the field of asthma regulation, interestingly, Shen and collaborators observed different clusters of Treg cells, one of which may play a role in allergic asthma pathogenesis through CCR6^+^ cell recruitment in the lungs of patients with allergic asthma [27]. These Treg cells migrate to the airways via CCR6 but fail to attenuate allergic inflammation, representing a pro-inflammatory Treg population during asthma exacerbations in humans.

Otherwise, in pediatric obese asthma, RNA-seq has identified CD4^+^ T cell subtypes programmed for *CDC42* upregulation, T1-mediated inflammation, and steroid resistance, contributing to the obese asthma phenotype and demonstrating non-atopic T cell responses (pre-print results [28]). Additionally, scRNA-seq on CD4^+^ T cells from peripheral blood of obese asthmatic patients revealed significant overrepresentation of interferon-related signaling pathways, driven by interferon-stimulated genes. These findings suggest that CD4^+^ T cells in obese asthmatics have been polarized towards a T1 response, with *EIF2AK2* as a potential treatment target for low-type 2 obese asthmatics [29]. Finally, Helou et al. characterized the role of Pd-1 in a clinically relevant mouse model of neutrophilic airway hyperresponsiveness, demonstrating that the absence of Pd-1 in Cd4^+^ T cells significantly induces exacerbations, airway hyperresponsiveness, and lung inflammation [30]. Their scRNA-seq data further supported the preclinical therapeutic potential of PD-1 agonists in neutrophilic asthma.

## 3. ScRNA-Seq Studies of Eosinophils

Eosinophils develop in the bone marrow from CD34^+^ stem cells and express the IL-5 receptor (IL-5R) prior to being released into the bloodstream. They may either remain in the bone marrow or migrate to tissues, where they help maintain plasma cells and regulate metabolic homeostasis [31], or they are involved in allergic diseases by the action of several cytokines, such as IL-5, which is crucial for eosinophil development, facilitating maturation and mobilizing eosinophils to sites of allergic inflammation [1]. Once recruited, eosinophils release proinflammatory cytokines, chemokines, cytotoxic proteins, lipid mediators, and Th2 cytokines, leading to lung tissue damage, airway remodeling, and bronchial hyperresponsiveness [32,33]. They also form extracellular traps linked to Charcot–Leyden crystals, which affect mucus viscosity and complicate its expulsion [34].

Eosinophils can be used as biomarkers for diagnosing eosinophilic asthma, with serum or sputum eosinophil counts often employed to monitor treatment response, particularly to biologics like dupilumab [35]. In fact, serum eosinophil count is a key indicator in determining the asthmatic endotype [2]. It is believed that there are two major eosinophil subpopulations: hypodense and normodense. Hypodense eosinophils, the active population, are sensitive to corticosteroids and found in elevated numbers in the blood and bronchoalveolar lavage of asthmatic patients. In contrast, normodense eosinophils reside in tissues when there is no inflammation [36].

ScRNA-seq is a powerful tool for comparing eosinophils across different tissues at a genomic level. It holds the potential to demonstrate human eosinophil subpopulations, as evidenced by studies in blood [37], bone marrow [38], esophagus [39], and nasal polyps [40]. However, this remains a topic of ongoing debate. For example, Rodrigo-Muñoz et al. did not find significant differences in eosinophil markers between healthy and asthmatic patients [37], while Ben-Baruch Morgenstern et al. identified two eosinophil populations in the esophagus: one resembling circulating eosinophils and another larger population expressing cytokine-related genes [39].

Additionally, Jorssen et al. applied scRNA-seq to understand the role of IL-5 in eosinophil maturation from bone marrow progenitor cells [38], while Iwasaki et al. used this technique to track gene expression related to nasal polyp development and eosinophil population heterogeneity in chronic rhinosinusitis [40].

Challenges in this field include limited sample sizes, low RNA content in eosinophils, and small numbers of eosinophils in certain tissues. Future studies should focus on larger, paired sample sets from patients with varying asthma phenotypes and endotypes to better understand eosinophil heterogeneity [37]. Improved data analysis techniques and solutions for RNA loss will enhance the quality of results.

## 4. ScRNA-Seq Research of Epithelial Cells

Epithelial cells serve as the first line of defense in the respiratory tract and lungs, acting as a physical, chemical, and immunological barrier against inflammatory stimuli and antigens. Their activation is closely associated with allergic sensitization, a key feature of asthma [41]. Upon damage or exposure to pro-inflammatory stimuli, epithelial cells secrete molecules such as IL-25, IL-33, and TSLP, referred to as alarmins, which orient the immune response towards a T2-type response [42]. In addition, epithelial goblet cells, which specialize in mucus secretion, undergo hypersecretion during asthma progression, contributing to one of the characteristic features of asthma: excessive mucus production. On the other hand, the role of airway epithelial cells in non-T2 asthma has been described [43]. There is a wide array of mechanisms within the T2 asthma phenotype that differentially impact the epithelial cells. These mechanisms include increased mucus production, degradation of tight junctions, resulting in greater barrier permeability, morphological alterations, changes in stem cell numbers, and elevated cytokine and chemokine synthesis (such as CCL11/Eotaxin, CCL2/MCP1, CXCL8/IL-8, and CXCL10/IP-10). Therefore, understanding these cells is essential for identifying the triggers of the T2 response in asthma and their role in airway remodeling.

ScRNA-seq not only allows the detailed description of molecular cell phenotypes but also helps predict cell–cell interactions and cell state transitions. For example, the application of this technology in mouse lung tissue has led to the discovery of a novel airway epithelial cell type called ionocytes, identified by their expression of V-ATPase subunits (*Atp6v1c2* and *Atp6v0d2*) and *Cftr* genes [44,45].

Although relatively few studies have focused on epithelial cells using scRNA-seq, those that have been conducted provide valuable insights into asthma and the T2 inflammatory response. A few years ago, RNA-seq studies revealed significant heterogeneity among epithelial cells in the human lung, identifying at least 10 different epithelial cell populations in the upper and lower airways as well as in the parenchyma [16]. This study also identified a mucous ciliated cell state in the airway epithelium of asthmatic patients, contributing to mucus cell metaplasia. In particular, the gene expression profile of *FOXJ1*^+^ cells resembled that of goblet cells, suggesting that T2 cytokines induce a goblet-like state in ciliated cells, further supporting mucous cell hyperplasia and metaplasia. Both phenomena contribute to the increased number of mucin-producing cells observed in asthma. In summary, the authors described the cellular landscape of the asthmatic airway wall and identified a unique, disease-associated airway epithelial cell state distinct from that of healthy individuals. Other studies on human airway cells aimed to explain two key aspects of asthmatic pathology: (i) the reprogramming of epithelial cells, leading to increased *MUC5AC* expression and mucus secretion, and (ii) the effects of IL-13 on epithelial cells, which are related to IL-13-induced asthma [46]. Using scRNA-seq, the researchers examined the whole-transcriptome response to IL-13 in different human airway epithelial cells. They found that, under the influence of IL-13, epithelial cells not only increased *MUC5AC* expression but also altered their transcriptomes and functions, producing pathological secretions with new mucus constituents and reduced homeostatic defense proteins. Furthermore, IL-13 induced metaplastic events by modulating the expression of several genes involved in mucus secretion, including *MUC5AC*, *MUC2*, *ITLN1*, and *ITLN2* [47,48]. Additionally, the study revealed that IL-13 causes a pan-epithelial loss of innate airway defenses, particularly those related to secreted detoxification and antimicrobial genes, thereby increasing the susceptibility of allergic asthmatic patients to respiratory infections.

Moreover, scRNA-seq has highlighted the role of specific genes in epithelial cells and their potential implications in asthmatic pathology. For instance, a recent study by Qiao and colleagues developed with data from asthmatic patients demonstrated the role of *STEAP4* using scRNA-seq [49]. Previous research has shown that STEAP4 inhibits inflammation by blocking inflammatory cytokines such as IL-6 and IL-8, reducing the expression of pro-inflammatory cytokines (TNF-α and MCP-1), and increasing levels of the anti-inflammatory cytokine IL-10 [50]. In their study, Qiao et al. revealed an upregulation of the MIF pathway in samples with low *STEAP4* expression in asthmatic patients, suggesting that STEAP4 may exert an anti-inflammatory effect through downstream signaling pathways in airway epithelial cells.

## 5. Single-Cell Transcriptomic of Type 2 Innate Lymphoid Cells

Group 2 innate lymphoid cells (ILC2s) are part of the innate immune system, distinguished by their lack of antigen receptors but functional similarity to Th2 cells. ILC2s secrete type 2 cytokines, including IL-4, IL-5, and IL-13, and are essential for maintaining mucosal homeostasis. They also play key roles in various pathological conditions, such as bronchial asthma, chronic rhinosinusitis, atopic dermatitis, and organ fibrosis [51,52,53,54,55].

The emergence of scRNA-seq has significantly advanced our understanding of the development and interaction of diverse cell populations, enabling the study of gene expression across individual cells at a genome-wide scale [56]. This technology has revealed that ILCs originate from common lymphoid progenitors, which differentiate into distinct ILC lineages [57,58]. For example, a study by Yong Yu et al. used scRNA-seq to analyze ILC precursors in mouse bone marrow, uncovering differentiation pathways of ILC1s, ILC2s, and ILC3s from a common Pd-1-expressing precursor. The development of ILC2s was found to require Bcl11b and IL-25 receptor expression, with activated ILC2s re-expressing PD-1, which plays a key role in promoting cytokine production during influenza infection and acute lung inflammation [59]. Further studies using scRNA-seq and flow cytometry have demonstrated in a mouse model that the transcription factor Gata3 is crucial for ILC2 development, from early innate lymphoid progenitors to mature ILC2s [60,61,62].

ILC2s not only contribute to mucosal homeostasis but also initiate pathological inflammation in allergic asthma. However, the signals that direct ILC2s towards homeostasis or inflammation remain unclear. To identify these molecular signals, researchers performed scRNA-seq in ILC2s under steady-state conditions and after stimulation with alarmin cytokines, such as IL-25 and IL-33. This analysis revealed transcriptionally heterogeneous ILC2 subpopulations distinguished by proliferative, homeostatic, and effector gene expression. Authors suggested that scRNA-seq can identify this diversity [63,64], both when changes in cell states are continuous across a population [63] and when there are discrete subpopulations of varying sizes, including in intestinal ILCs [65].

Single-cell transcriptomics has also been used to examine the immune landscape during steroid-resistant asthma exacerbations. Using a mouse model of HDM- and LPS-induced asthma following dexamethasone treatment, researchers identified 20 major immune cell subsets with distinct gene expression profiles. Notably, the expression of *Il-4* and *Il-13* by basophils, ILC2s, and Cd8^+^ memory cells was found to be largely resistant to steroid treatment [66].

scRNA-seq has further revealed insights into ILC responses during COVID-19 infection. In COVID-19 patients, the frequencies of both ILC1 and ILC2 cells significantly increased. Differentially expressed genes were associated with viral response and ILC proliferation, activation, and homeostasis. In addition, gene regulatory network analysis identified ILC-specific regulons that drive differential gene expression in each subset, contributing to the immune response of the organism to the virus [67].

Asthma exacerbation in women often correlates with fluctuations in ovarian hormones, with 30–40% experiencing worsened symptoms around menstruation. This suggests a role for hormonal changes in asthma progression. In a study developed by Trivedi et al., scRNA-seq was used to investigate the effects of ovarian hormones on ILC2s and the NF-κB signaling pathway following OVA sensitization and challenge [68]. The study found differentially expressed genes in lung ILC2s from male and female mice, shedding light on the suppressive effects of estrogen on ILC2s, which may offer protective benefits in female asthmatics.

The interactions between immune cells and other physiological systems, such as the nervous system, are also critical in allergic inflammation. In an intestinal immune cell atlas generated using scRNA-seq, Xu et al. identified that the neuropeptide α-CGRP regulates ILC2 responses and the allergic reaction [69]. This study highlighted a neuro-immune cellular circuit that modulates T2 inflammation, emphasizing the role of α-CGRP in maintaining ILC2 homeostasis and immune response. Additionally, Xu and colleagues combined scRNA-seq with genetic and physiological manipulations to examine type 2 intestinal inflammation. Their computational analysis revealed shifts in cell compositions from mice and programs in response to inflammation, with a notable increase in *α-Cgrp* transcription in ILC2s [69].

Though ILCs represent a small fraction of the immune cells of the lung, they play crucial roles in early responses to pathogens and in promoting adaptive immunity, particularly in asthma progression [51,52,53,54,55]. Single-cell transcriptomic studies of lung immune cells provide deeper insight into the cellular composition of the lung during asthma exacerbation and steroid resistance, contributing to a detailed immune cell atlas that aids our understanding of cellular mechanisms of asthmatic disease [18].

## 6. ScRNA-Seq Studies in Other Immune Cells

### 6.1. Dendritic Cell

As mentioned earlier, scRNA-seq is a useful and novel technique that has been applied in the study of the pathogenesis of asthma and other allergic diseases, specifically targeting immune cells. In this context, studies have revealed different subsets of DCs, macrophages, and other immune cells, such as B lymphocytes.

DCs are the most potent antigen-presenting cells in the immune system, serving as a crucial link between innate and adaptive immunity. In allergic asthma, specific DC subsets are pivotal in initiating and sustaining T2 immune responses triggered by allergens in the airways [70]. They are a heterogeneous group of cells with various subtypes that differ in phenotype, function, and localization [71]. Based on lineage-defining transcriptional programs and functional characteristics, conventional cDCs are classified into two main populations: type 1 (cDC1) and type 2 (cDC2) [72]. However, in-depth analysis could reveal additional subtypes. In this context, scRNA-seq analysis has demonstrated the existence of two constitutively present *Irf8*^+^ and *Batf3*^+^ conventional cDC1 clusters in the murine lung, both of which play roles in tolerance induction and inflammation [73]. On the other hand, a recent study conducted in an allergic HDM mouse model demonstrated, using scRNA-seq, several subpopulations of mouse cDC2, including a non-migratory lung-resident population that primarily stimulates allergen-specific Th17 cell differentiation [74]. The study identified five distinct clusters of cDC2, which differed in their maturation status and ability to induce differentiation into different T helper cell subsets. The *Lyc6*^+^ cDC2 cluster, after allergen exposure, accumulates in the lung and promotes Th17 differentiation, while *Cd200*^+^ mature cDC2 induces Th2 but not Th17 differentiation. Moreover, it was observed in both mouse and human studies that C1q is secreted by the *Csf1r1*^+^ cDC2 subset within cDCs, suggesting that the C1q-LRP1 axis could serve as a potential target for translational therapies aimed at preventing and controlling allergic lung inflammation, as the depletion of C1q in conventional DCs significantly attenuates features of asthma [75].

Currently, another study is being developed in pediatric asthmatic children and healthy controls, utilizing scRNA-seq in peripheral blood mononuclear cells (PBMCs) to investigate the cellular etiology of childhood asthma (pre-print results, [76]). The authors showed, using scRNA-seq, that DCs had the largest number of both incoming and outgoing interactions among other immune cells, likely functioning as a signaling hub in cell communication. Therefore, scRNA-seq phenotype and gene expression studies have revealed heterogeneity within the same DC subtype, reinforcing the notion that DCs have evolved to enhance the flexibility of the immune system, enabling it to respond effectively to a broad spectrum of threats.

### 6.2. Macrophages

On the other hand, macrophages are another cell type involved in asthma pathogenesis. Lung macrophages play a role in allergic airway inflammation by facilitating the recruitment and activation of inflammatory cells, as well as by secreting factors that promote thickening and remodeling of structural cells [77]. An scRNA-seq study revealed a new gene signature of *FCN1*^+^ macrophages in sputum samples from subjects with eosinophilic asthma, which could be a potential therapeutic target in this type of asthma [78]. In this study, the authors identified that *FABP4*^+^ macrophages were the largest subset of sputum macrophages. Moreover, they found increased gene expression in *SPP1*^+^ macrophages, which was linked to antigen processing and presentation, response to bacterial molecules, and macrophage activation. This suggests that *SPP1*^+^ macrophages may have a classical role in the innate immune response during eosinophilic airway inflammation.

### 6.3. B Lymphocytes

B cells, as pivotal components of the immune system, play a significant role in the pathogenesis of asthma. They are primarily recognized for their ability to produce IgE, which triggers the allergic response. Some studies involving scRNA-seq have explored B cells in the context of asthma. For instance, Guo et al. described in humans that *C1orf64* and *C7orf26* were potential key genes in asthma pathogenesis, found in T, B, and natural killer cells. Their dysregulation could alter inflammatory responses and contribute to asthma pathogenesis [79]. In another study focused on equine neutrophilic severe asthma, scRNA-seq was performed on bronchoalveolar lavage (BALF) cells collected from six asthmatic and five control horses. The study identified six major clusters, including B cells, T cells, monocytes/macrophages, dendritic cells, neutrophils, and mast cells [80]. The authors highlighted that BALF from asthmatic horses was enriched in B cells, in addition to neutrophils, which have the potential to differentiate into plasma cells and become activated. Moreover, these B cells from asthmatic horses showed increased expression of *POU2AF1* and deregulated *YBX3*, which could be associated with lung dysfunction similarly to humans [81,82,83]. Aranda et al. performed scRNA-seq on PBMCs, identifying a subpopulation of human IgG memory B cells with a signature characterized by the expression of *FCER2/CD23*, *IL4R*, *IL13RA1*, and *IGHE* (all of which are recognized targets of IL-4/IL-13 signaling in B cells) [84]. Moreover, they found that the frequency of these cells was higher in asthmatic patients compared to non-atopic healthy subjects, and there was a positive correlation between their frequency and the levels of IgE antibodies found in plasma.

Additionally, scRNA-seq was performed in spleen B cells from mice, mouse lung cells, and human lung parenchymal cells. Several B cell subsets were identified, including B cells secreting IL-10 [85]. In these B cell subsets, *Foxo1* was differentially expressed, showing that *Foxo1* inhibits the development of IL-10-producing B cells by suppressing *Blimp-1* expression (which is essential for allergen-induced asthma and Th2 cell development in the lung) [86], and thus contributing to asthma immunopathology. Three clusters of B cells (BC, pro-B cells, and plasma B cells) were identified in an HDM-induced asthma mouse model, based on the expression of *Cd79a*, *Ifi30*, and *Jchain* [87]. The authors described that plasma B cells were significantly increased following exposure to HDM, highlighting the role of IgE-producing plasma B cells in T2-mediated inflammation [88].

## 7. Conclusions and Future Perspectives

Asthma is a complex and heterogeneous disease with a pathogenesis that is affected by several factors. Previous asthma transcriptomic studies have been limited in their understanding of the cellular and molecular composition of the lung immune response by the lack of adequate tools to study differences between cells that are histologically indistinguishable.

The arrival and rapid development of scRNA-seq technology has made it possible to study individual cells in tissues in depth, classify these cells into cell types, and characterize variations in their molecular profiles. ScRNA-seq has considerably expanded the understanding of asthma by uncovering detailed aspects of cellular heterogeneity within the lungs and immune system that were previously inaccessible through bulk RNA-seq. Among the most significant findings from scRNA-seq studies is the discovery of distinct immune cell subsets implicated in asthma pathogenesis, including specific T cell subtypes and ILC2. These advances have deepened our understanding of T2 and non-T2 asthma endotypes, elucidating differences in inflammatory pathways at the cellular level. The currently available literature about tissue scRNA-seq has increased the understanding of lung and other tissue cell composition and provided a comprehensive and important source of data for constructing a cell atlas with data about functions of cell types and interactions altered in the development of asthma.

RNA-seq is a highly useful tool for personalized asthma diagnosis and treatment, presenting significant potential advantages that could be leveraged in routine clinical practice. The discovery of new pathways and cellular markers could help to identify new asthma endotypes, involving not only novel cell subsets but also distinct biological mechanisms and the understanding of regulatory gene networks that predict immune function. On the other hand, it could reveal new signaling pathways that may serve as therapeutic targets for asthma treatment. For example, using this approximation, several identified genes have been associated with cytokines (e.g., *IL-9*, *IL-13*), receptors (e.g., *IFNR*, *CXCR4*), and signaling pathways (e.g., Egfr).

Regarding clinical implementation, this technique is still far from being fully achievable. Its high cost and the lack of standardized protocols present significant challenges to short-term application. Additionally, result analysis requires trained personnel, adding another layer of complexity. However, some solutions being investigated to address these challenges include improved sequencing technologies, up-scaled sequencing, and multiplexing. Additionally, some companies are developing specific kits for scRNA-seq that facilitate standardization and simplify the process for clinical laboratories. The automation of data interpretation, such as artificial intelligence, is also a key development; the creation of algorithms that can help interpret results and translate them into useful clinical information could reduce the burden on specialists and accelerate the diagnostic process. Therefore, further studies and advancements in this technique are essential to make it clinically applicable, as it holds considerable promise for the future.

Despite the maturity of technology and its advantages, it has some limitations (Table 1). The preparation of tissue single-cell suspensions is complicated; some single-cell isolation technology may have an impact on cell integrity, resulting in data distortion or loss, and the limits on the number of cells that can be sequenced can hamper the identification of rare cells. Another complication is the high cost of scRNA-seq technology and the need for trained bioinformaticians to analyze scRNA-seq.

Currently, there are only a limited number of scRNA-seq studies on humans with asthma; more studies should be conducted and extended to single-cell multi-omics sequencing technology to improve the understanding of the pathogenesis of asthma.

## Figures and Tables

**Table 1:** Advantages and limitations of scRNA-seq.

Advantages	Limitations
Single-cell resolution	Higher technical complexity
Allows analysis of cellular heterogeneity	Requires specialized equipment and platforms
Detects differences in gene expression between cells within the same tissue	High cost
Identifies rare or transitioning cell populations	Generation of large data volumes
Helps identify cell subpopulations	Data interpretation can be challenging due to complexity
Allows detailed transcriptomic profiling	Limited sequencing depth
Enables study of specific cellular states and dynamics	Amplification artifacts (over-amplification or bias)
Useful in studying complex diseases, such as asthma	Challenges in normalization and sample comparison
